# Effects of Wine and Its Microbial-Derived Metabolites on Intestinal Permeability Using Simulated Gastrointestinal Digestion/Colonic Fermentation and Caco-2 Intestinal Cell Models

**DOI:** 10.3390/microorganisms9071378

**Published:** 2021-06-24

**Authors:** Irene Zorraquín-Peña, Diego Taladrid, Alba Tamargo, Mariana Silva, Natalia Molinero, Dolores González de Llano, Begoña Bartolomé, M. Victoria Moreno-Arribas

**Affiliations:** Institute of Food Science Research, CIAL (CSIC-UAM), C/Nicolás Cabrera 9, 28049 Madrid, Spain; irene.zorraquin@csic.es (I.Z.-P.); d.taladrid@csic.es (D.T.); alba.tamargo@csic.es (A.T.); mariana.silva@csic.es (M.S.); natalia.molinero@csic.es (N.M.); d.g.dellano@csic.es (D.G.d.L.); b.bartolome@csic.es (B.B.)

**Keywords:** wine polyphenols, tight junctions, simulated digestion, microbiota, gut permeability

## Abstract

This paper explores the effects of wine polyphenols on intestinal permeability in in vitro conditions. A red wine (2500 mg/L of gallic acid equivalents) was sequentially subjected to gastrointestinal and colonic digestion in the Dynamic Gastrointestinal Simulator (simgi^®^) to obtain two simulated fluids: intestinal-digested wine (IDW) and colonic-digested wine (CDW). The two fluids were incubated with Caco-2 cell monolayers grown in Transwell^®^ inserts, and paracellular permeability was measured as transport of FITC-dextran. Non-significant decreases (*p* > 0.05) in paracellular permeability were found, which was attributed to the relatively low phenolic concentration in the solutions tested (15.6 and 7.8 mg of gallic acid equivalents/L for IDW and CDW, respectively) as quercetin (200 µM) and one of its microbial-derived phenolic metabolites, 3,4-dihydroxyphenylacetic acid (200 µM), led to significant decreases (*p* < 0.05). The expression of tight junction (TJ) proteins (i.e., ZO-1 and occludin) in Caco-2 cells after incubation with IDW and CDW was also determined. A slight increase in mRNA levels for occludin for both IDW and CDW fluids, albeit without statistical significance (*p* > 0.05), was observed. Analysis of the microbiome and microbial activity during wine colonic fermentation revealed relevant changes in the relative abundance of some families/genera (i.e., reduction in *Bacteroides* and an increase in *Veillonella*, *Escherichia*/*Shigella* and *Akkermansia*) as well as in the microbial production of SCFA (i.e., a significant increase in propionic acid in the presence of IDW), all of which might affect paracellular permeability. Both direct and indirect (microbiota-mediated) mechanisms might be involved in the protective effects of (wine) polyphenols on intestinal barrier integrity. Overall, this paper reinforces (wine) polyphenols as a promising dietary strategy to improve gut functionality, although further studies are needed to evaluate the effect on the intestinal barrier under different conditions.

## 1. Introduction

The intestinal barrier is a dynamic structure separating the internal host from the gut lumen and is comprised of several items: mucus and epithelial layers, immunological (lymphocytes and innate immune cells) and humoral (defensins and immunoglobulin A (IgA) elements, and muscular and neurological elements [1]. Of special importance for the integrity of the intestinal barrier are the tight junctions (TJ) formed between neighboring epithelial cells, including several proteins such as occludins, claudins, junctional adhesion molecules, and plaque proteins [2]. One of the main properties of the intestinal barrier is what is defined as intestinal permeability (IP), which selectively allows or restricts the exchange of water, ions, and macromolecules between the intestinal lumen and the underlying tissues. This exchange occurs through both transcellular pathways, governed by the cell-specific profile of transporters and channels, and paracellular pathways, mainly controlled by tight junctions [3]. All these components are interconnected with the intestinal microbiota, forming a sophisticated and dynamic biological system affecting IP [4]. Moreover, the disruption of the intestinal barrier results in what is known as “leaky gut” and has been related to inflammation processes and intestinal dysfunction, leading to the appearance of autoimmune and intestinal diseases such as coeliac disease [5], colon cancer [6], or inflammatory bowel disease (IBD) [7]. In the case of IBD, alterations of the TJ proteins are a key matter, although it is not clear whether these findings are primary or secondary to the pathogenesis of the disease [8].

Dietary components such as fiber and polyphenols might counteract inflammation processes and intestinal dysfunction, with their biological action being mediated through the gut microbiota [9]. Polyphenols are plant secondary metabolites, chemically characterized by a benzene ring substituted by one or several hydroxyl groups (-OH). According to their chemical structure, they are divided into two large groups of compounds: non-flavonoids and flavonoids. Present in relatively high quantities in numerous plant-derived foods and beverages, it has been suggested that polyphenols promote gut health and prevent pathologies associated with the impairment of the intestinal barrier [10]. Thus far, most of the studies concerning the effects of polyphenols on IP have been carried out in cell models, such as Caco-2 and HT-29 cell lines, prior to more advanced animal models [11]. In spite of the intense current research on this matter, the mechanisms behind the protective effects of polyphenols on intestinal barrier integrity have not been fully clarified. A recent review about polyphenols and IP [1] tentatively categorized polyphenol activities into three levels: (1) intraluminal level, by modulating microbiota composition, endotoxin and/or short-chain fatty acid (SCFA) production, redox status, and dietary component absorption and/or activity; (2) intracellular level, by regulating the expression of TJ, AJ, GJ, and desmosome proteins, upregulating kinases and Nrf-2, and downregulating NF-κB and TLR4; and (3) systemic level, by maintaining the functional immune system and controlling inflammatory processes in general.

Wine is probably one of the foods that shows greater and more diverse phenolic content composition. Within wine phenolic compounds there are flavonoid compounds such as flavan-3-ols, flavonols, flavones, and anthocyanins (only in red wine), and non-flavonoid compounds such as hydroxybenzoic acids, hydroxycinnamic acids, stilbenes and hydrolyzable tannins. To the best of our knowledge, so far, only two studies have evaluated the effect of grape/wine polyphenols on intestinal permeability in vitro. Nunes et al. [12] reported the effects of a red wine extract on paracellular permeability and TJ protein expression in cytokine-stimulated HT-29 cells. Nallathambi et al. [13] investigated the effects of a proanthocyanidin-rich grape seed extract on TJ in Caco-2 cells previously treated (or not treated) with bacterial lipopolysaccharide (LPS) to induce oxidative stress. In both cases, grape/wine polyphenols promoted favorable changes in different markers of intestinal barrier integrity. However, the extracts were used directly without previous intestinal digestion, obviating the fact that polyphenols are extensively metabolized during their passage through the gastrointestinal tract.

To date, there are some studies that evaluate the biotransformation of polyphenols into different matrices through gastrointestinal simulators such as the SHIME (Simulator of Human Intestinal Microbial Ecosystem) and the simgi^®^ (Dynamic gastrointestinal simulator). For example, in the study by Freire et al. [14], SHIME was used to digest fermented goat milk with grape juice and grape pomace extract, and an increase in SCFA and antioxidant capacity was observed. Another study in which the simgi^®^ was fed with red wine resulted in an increase in the phenolic metabolites produced, as well as an increase in SCFAs and in the microbial genera *Lactobacillus* and *Bacteroides* [15].

Based on this background, the aim of this paper was to study the in vitro effect on intestinal permeability of wine polyphenols after being subjected to simulated gastrointestinal digestion. For that, the dynamic gastrointestinal simulator (simgi^®^) was fed with a red wine to obtain an intestinal-digested fluid (intestinal-digested wine, IDW) that underwent further colonic fermentation to lead to a colonic-digested fluid (colonic-digested wine, CDW). As an indicator of gut barrier functionality, a Caco-2 intestinal epithelium model under homeostatic conditions was used to assess the effect of the digested-wine fluids on paracellular transport and TJ protein expression. In parallel, standards of quercetin, a flavonol present in wine, and its microbial-derived metabolite 3,4-dihydroxyphenylacetic acid (3,4-dhpa) were assayed. As potential indirect mechanisms for influencing IP, changes in the microbiome as well as in microbial metabolic activity (i.e., SCFA or phenolic metabolites production) during wine colonic fermentation were determined.

## 2. Materials and Methods

**Figure 1:**
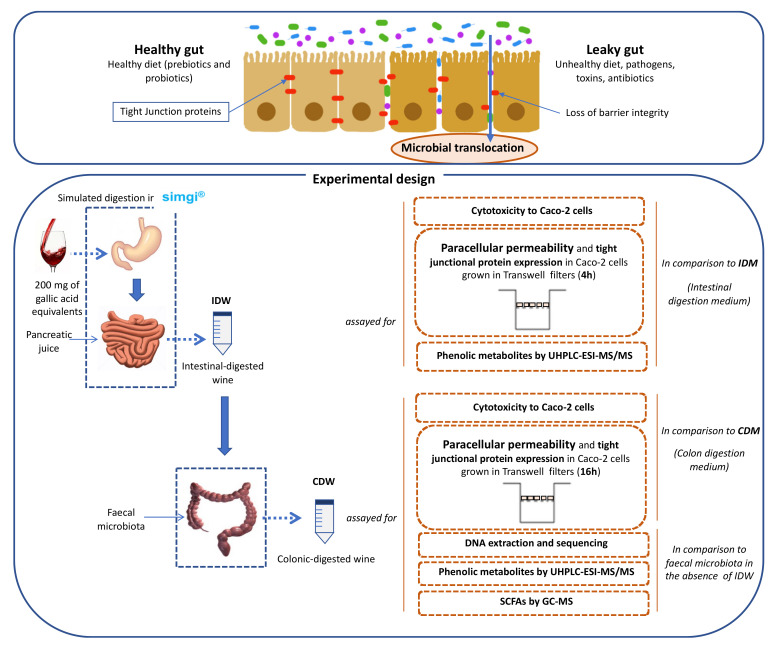
Scheme for the obtention of digested-wine fluids (IDW and CDW) and the study of their effects on intestinal permeability and gut microbiota.

Below is a diagram of the experimental work scheme carried out in this work, including the different steps: gastrointestinal simulation/obtention of the digested samples, and experimental methodologies applied to the digested samples related to intestinal permeability (Figure 1).

### 2.1. Wine

A reserve red wine (Cabernet Sauvignon and Cabernet Franc, vintage 2006) was kindly provided by Bodegas Miguel Torres S.A. (Catalonia, Spain). The ethanol content in the wine was 13.5% and the total phenolic content reached 2500 mg of gallic acid equivalents/L. The detailed phenolic composition of the wine is presented in Table 1.

### 2.2. Simulated Digestion of the Wine

Simulated digestion of the wine was carried out in the dynamic SIMulator of the GastroIntestinal tract (simgi^®^) which was previously validated and optimized [15]. The simgi^®^ comprises five interconnected compartments that simulate the stomach, small intestine, ascending colon, transverse colon, and descending colon regions that can operate jointly or independently. In the present study, the system operated only with the stomach and small intestine compartments to simulate the dynamic gastrointestinal digestion. For that, two simulated juices were initially prepared: gastric juice and pancreatic juice. The simulated gastric juice consisted of pepsin (2000 U/mL) (Sigma-Aldrich, MERK, Kenilworth, NJ, USA) dissolved in simulated gastric fluid (SGF) [16]. The simulated pancreatic juice consisted of Oxgall Dehydrated Fresh Bile (6 g/L) (Difco™ BD, Franklin Lakes, NJ, USA) and pancreatin (4.24 g/L) (Sigma-Aldrich, MERK, USA) dissolved in simulated intestinal fluid (SIF) [16], and the solution was filtered by a polyethersulfone 0.45 µm-pore membrane. Initially, the simgi^®^ was preconditioned at 37 °C, and the stomach compartment was filled with 65 mL of SGF [16], while the small intestine compartment was filled with 55 mL of SIF [16]. Then, the system was fed with 80 mL of wine (200 mg of gallic acid equivalents). In the gastric phase, peristalsis was set to 10 s^−1^, the pH curve was gradually lowered from 5.6 to 1.8 by the total addition of 6 mL of 0.5 M HCl, and simulated gastric juice was released by a flux of 3.9 mL/min up to a total volume of 15 mL. To simulate physiological gastric emptying, flow transfer from the stomach compartment to the small intestine compartment was automatically programmed by the Elashoff function [17]. Following this function, a total of 95 mL of the efflux from the stomach was gradually transferred to the small intestine compartment. Simulated pancreatic juice (40 mL) was also added to the small intestine compartment at a 5 mL/min flow rate. Then, intestinal digestion was carried out for 2 h at 37 °C, pH 7 and 150 rpm under anaerobic conditions. At the end of this phase, the intestinal-digested wine (IDW) was obtained, resulting in an approximate dilution of 1:4 (*v*/*v*) in respect to the initial wine. Part of this fluid was reserved for subsequent colonic fermentation (Figure 1). An aliquot of this IDW fluid was centrifuged at 10,000 rpm for 10 min at 4 °C, and the pellet was frozen and kept at −80 °C. The supernatant was aliquoted and kept at −80 °C for further analysis.

In a second phase, the IDW fluid was subjected to static fermentations with fecal microbiota obtained from a healthy volunteer who had made sure they had not consumed any antibiotics during the 6 months prior to the sample collection. Three different sterilized fermentation flasks were filled with 25 mL of colonic nutrient medium [18] and 30 mL of IDW, then inoculated with 5 mL of the fecal suspension (1 g of feces in 10 mL of 0.1M PBS, pH 7), prepared as previously described [19]. Colonic fermentations were carried out simulating the conditions of the distal region of the human large intestine: pH 6.8, 37 °C and an anaerobic atmosphere for a period of 48 h [18]. An identical procedure replacing IDW with 30 mL of colon nutrient medium, also in triplicate, was carried out as fermentation control. Samples were collected at 0, 24 and 48 h. The ones resulting from 48 h of incubation were named “colonic-digested wine” (CDW) (Figure 1). Samples for further metagenomic analysis were centrifuged at 10,000 rpm for 10 min at 4 °C and pellets were frozen and kept at −80 °C. Supernatants were aliquoted and kept at −80 °C for further analysis.

### 2.3. Caco-2 Cells Culture

Human epithelial colorectal adenocarcinoma cells (Caco-2 cells) (ATCC^®^ HTB-37TM) were used as a cellular model. They were cultured in high-glucose Dulbecco’s Modified Eagle’s medium (DMEM) (Biowest, Nuaillé, France) supplemented with a 1% (*v*/*v*) penicillin/streptomycin solution (Sigma-Aldrich, St. Louis, MO, USA), a 10% (*v*/*v*) heat-inactivated foetal bovine serum (FBS) solution (Biowest, Nuaillé, France) and a 1% (*v*/*v*) non-essential amino acid solution (Lonza, Basel, Switzerland). Cells were maintained at 37 °C in 5% CO_2_ atmosphere and constant humidity.

### 2.4. Cytotoxicity Assay

Aliquots of IDW and CDW fluids were defrosted and diluted (1:4, 1:10 and 1:40 *v*/*v*) with culture medium without FBS, and filtered through a 0.22 µm filter (Symta, Madrid, Spain). The cellular toxicity against proliferating Caco-2 cells of the diluted fluids was measured using the colorimetric 3-(4,5-dimethylthiazol-2-yl)-2,5-diphenyltetrazolium bromide (MTT) assay. Briefly, Caco-2 cells, seeded the previous day at a density of 3.6 × 10^5^ cells/mL (100 µL/well), on 96-well plates were incubated with cell culture medium without FBS (control) or with diluted IDW and CDW fluids (1:4, 1:10 and 1:40, *v*/*v*) for 4 or 24 h. Then, the supernatants were replaced by MTT reagents (0.5 mg/mL) (Sigma-Aldrich, St. Louis, MO, USA). After 3 h of incubation, the MTT reagent was removed from the wells and 100 µL of dimethyl sulfoxide (DMSO, Sigma-Aldrich, St. Louis, MO, USA) was added to dissolve formazan crystals. Absorbance at 570 nm was measured on a Multiskan plate reader (Thermo Scientific, Waltham, MA, USA). Control absorbance was considered 100% cell viability and the results were expressed as the percentage of cell viability relative to untreated control cells. Assays were carried out in triplicate and three independent experiments were performed.

### 2.5. Paracellular Permeability Assay

Caco-2 cells grown at confluence were seeded at 1.5 × 10^5^ cells/mL in polycarbonate Transwell^®^ inserts (12 mm Ø, 0.4 µm pore size) (Costar; Corning Incorporated, Kennebunk, USA) and cultured as indicated above. After renewing the medium every 3 days, transepithelial electrical resistance (TEER) was measured with an Epithelial Volt/Ohm Meter (World Precision Instruments, Sarasota, FL, USA) to ensure that the cells had reached confluence and differentiation, which occurred at 21 days.

Differentiated cells were incubated with the digested-wine fluids (IDW or CDW) and pure phenolic compounds in two independent assays. In a first experiment, 500 µL of sample [the intestinal digested extract (IDW) or the simulated pancreatic juice (IDM) (both 1:40 *v*/*v* diluted in DMEM), or a quercetin solution (200 µM in DMEM), or DMEM (as control)] were added to the apical side of the Transwell^®^ inserts containing the Caco-2 cell monolayers. These plates were incubated at 37 °C for 4 h, simulating the time for which dietary compounds remain in the small intestine in in vivo conditions.

In a second experiment, in a similar way, 500 µL of sample colonic digested wine (CDW) or the colon nutrient medium (CDM) (both 1:40 *v*/*v* diluted in DMEM), or a 3,4-dyhydroxyphenylacetic acid (3,4-dhpa) solution (200 µM in DMEM), or DMEM (as control)] were added to cell monolayers and incubated for 16 h, which simulated the average time that dietary components remain in the colon. For both experiments, incubations were carried out in triplicate and experiments were repeated on three different days.

For both experiments, when the incubation time was over, the apical chamber solutions of the Transwell^®^ culture inserts were replaced by 500 µL of a DPBS (Dulbecco’s Phosphate-Buffered Saline) solution containing 1 mg/mL of fluorescein isothiocyanate (FITC-)-dextran 4 kDa and the basolateral sides of the plate inserts were filled with DPBS. Then, the plate was incubated at 37 °C for 30 min. Afterwards, 100 µL from the basolateral chamber of each well were taken in triplicate to measure the concentration of FITC-dextran by fluorescence intensity in a BioTek FL600 microplate reader (Biotek, Winooski, VT, USA) with an excitation wavelength of 480 nm and an emission wavelength of 520 nm.

### 2.6. TJ Protein Expression Assay

At the end of the experiments, Caco-2 cell monolayers were scraped and withdrawn with 500 µL of cold PBS, which was centrifuged at 1500 rpm for 10 min. Then, 350 µL of the RA-1 buffer (with 1% of β-mercaptoethanol) was added to the pellet prior to the mRNA extraction, which was carried out with a NucleoSpin^®^ RNA XS kit (Macherey-Nagel, Düren, Germany) following the manufacturer’s instructions. cDNA was obtained with a qPCRBIO cDNA Synthesis Kit (PCR Biosystems, Wayne, PA, USA) according to the procedure described by the manufacturer.

Finally, qRT-PCR was performed employing a ViiA™ 7 Real-Time PCR System (Applied Biosystems, Foster City, CA, USA) to quantify the genetic expression of the TJ proteins ZO-1 and occludin using the GADP gene as housekeeping. For that, 1 µL of cDNA was amplified in a 10 µL PCR reaction containing 0.5 µL of each primer (500 nM), 5 µL of PowerUp SYBR Green Master Mix (Applied Biosystems) and 3 µL of RNase/DNase-free water. PCR began with a first cycle at 95 °C for 3 min followed by 55 cycles, each composed of a denaturation step (95 °C, 10 s), an annealing step (55 °C, 30 s) and an elongation step (72 °C, 30 s). The primers employed in the amplification were as follows: ZO-1 F, 5′-GGTGAAGTGAAGACAATG-3′; ZO-1 R, 5′-GGTAATATGGTGAAGTTAGAG-3′; occludin F: 5′-ATGAGACAGACTACACAACTGG-3′; occludin R: 5′-TTGTATTCATCAGCAGCAGC-3′; GADPH F: 5′-TGCACCACCAACTGCTTAGC-3′; GADPH R: 5′-GGCATGGACTGTGGTCATGAG-3′. A melting curve was employed to ensure the specificity of the amplification products. The mRNA levels of each protein in the different samples were normalized against GADPH as a housekeeping gene and expressed as the fold increase with respect to their respective control using the E−ΔΔCT method.

### 2.7. Analysis of Phenolic Compounds by UHPLC-ESI-MS/MS

Prior to analysis, wine was filtered through a 0.22 μm filter (Symta, Madrid, Spain), and defrosted aliquots of IDW or CDW supernatants were filtered through a 0.22 μm filter (Symta, Madrid, Spain). The UPLC-ESI-MS/MS method for the determination of phenolic compounds was described in a previous study [20]. The multiple reaction monitoring mode (MRM) was followed to collect data. Quantification was carried out employing calibration curves of each corresponding standard compared to the internal standard (4-hydroxybenzoic-2,3,5,6-d4 acid). Detection was carried out by a triple quadrupole detector (TQD). The MS/MS parameters (cone voltage, collision energy and MRM transition) were described in a previous study [21]. Analysis was carried out in triplicate.

### 2.8. Metagenomics Analysis

Pellets from the fecal fermentations in the absence and presence of IDW were subjected to DNA extraction and 16S rDNA sequencing. Initially, several microbial counts were realized. Then, DNA was purified using the QIAamp DNA Stool Mini Kit (Qiagen, Hilden, Germany), following the manufacturer’s recommended protocol.

The two-step PCR Illumina^®^ protocol was used to prepare the libraries, including PCR Blockers in the first process for minimizing the amplification of mitochondrial and cloroplast DNA [22]. The V3-V4 region of the 16S ribosomal RNA gene was amplified by using the forward primer 5′-CCTACGGGNBGCASCAG-3′ and the reverse primer 5′-GACTACNVGGGTATCTAATCC-3′. Sequencing was subsequently carried out on an Illumina^®^ MiSeq instrument (Illumina^®^, San Diego, CA, USA) using 2 × 500 paired-end reads and then it was analyzed by amplifying and sequencing the V3-V4 region of the 16S rRNA gene. Raw files are available in the National Center for Biotechnology (NCBI) repository under project code PRJNA731070.

To denoise, align pairs and filter out chimeras in the raw data the DADA2 algorithm was employed [23]. The error correction model implemented in this algorithm allows the differentiation of even a single nucleotide, leading to the formation of amplicon sequence variants (ASVs). A total of 201,387 good-quality reads were obtained for bacterial DNA. The taxonomic assignment was performed using the naïve Bayesian classifier implemented in DADA2 using the Silva v132 database as a reference [24], with a bootstrap cut-off of 80%.

### 2.9. Analysis of Fatty Acids by GC-MS

Aliquots of CDW supernatants from the fecal fermentations in the absence and presence of IDW were defrosted and filtered through a pore size of 0.22 µm. Analysis of SCFAs was carried out following the SPME-GCMS method in accordance with the methodology described in a previous work [15]. The SCFA concentration was calculated from calibration curves of their corresponding standards compared to the internal standard (2-methylvaleric acid). Analyses were carried out in duplicate.

### 2.10. Statistical Analysis

All the statistical tests were carried out using R. Each treatment tested in Caco-2 cells was individually compared to the control incubation employing the T-test (vegan package) for both paracellular transport and TJ protein expression assays. The alpha diversity indices were calculated using the phyloseq package, and the “Heatplus“ and “gplots” packages were used to show the relative abundance of bacterial genera with more than 1% of abundance.

## 3. Results and Discussion

### 3.1. Effects of Digested-Wine Fluids on Paracellular Permeability

The toxicity on Caco-2 cells of different dilutions (1:4, 1:10 and 1:40, *v*/*v*) of the two digested-wine fluids (IDW or intestinal-digested wine, and CDW or colonic-digested wine) at two incubation times (4 h for IDW and 16 h for CDW) was initially evaluated (results not shown). Cytotoxicity was associated with the components of the media used in the simulated digestions (IDM or intestinal digestion medium, and CDM or colonic digestion medium) rather than the phenolic compounds themselves. Finally, for both fluids, 1:40 (*v*/*v*) dilutions were selected for further assessment of their effects on paracellular permeability as lower dilutions were harmful (>40% cytotoxicity) to cells.

**Figure 2:**
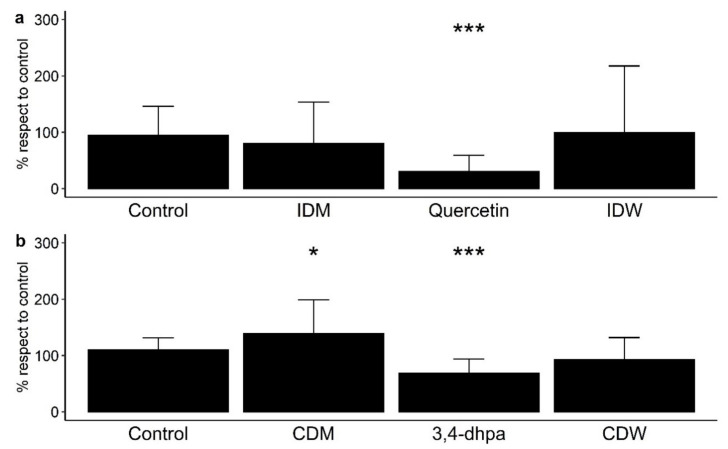
Effect on paracellular permeability in Caco-2 cells of (**a**) intestinal digestion medium (IDM), quercetin (200 µM), and intestinal-digested wine (IDW) after 4 h of incubation, and (**b**) colon digestion medium (CDM), 3,4-dihydroxyphenylacetic acid (200 µM) and colonic-digested wine (CDW) after 16 h of incubation. * significant differences to the control at *p* < 0.05, and *** *p* < 0.001.

Paracellular permeability across polarized Caco-2 monolayers was measured as means of the paracellular transport of FITC-dextran from the apical side to the basolateral side of the Transwell^®^ inserts (% with respect to control) (Figure 2). Note that Figure 2a shows the results from the determinations carried out in conditions that simulated the time that dietary compounds remain in the small intestine (4 h incubation with cells), and Figure 2b shows the results from those conditions in the colon region (16 h incubation). Each treatment tested in Caco-2 cells was statistically compared with the control (no treatment) to assess the effect of all the components of the fluids after digestion in the absence and presence of wine (Figure 2). At small intestine level, IDW and the corresponding intestinal digestion medium (IDM) exhibited non-significant effects (*p* > 0.05) on paracellular permeability with respect to the control (Figure 2a). Non-significant differences were either found between IDM and the corresponding intestinal digestion medium (IDM) (data not shown). However, quercetin (200 µM or 60 mg/L) significantly (*p* < 0.001) reduced paracellular transport of FITC-dextran (Figure 2a). In simulated colon conditions, the digested wine (CDW) slightly decrease paracellular permeability respect to the control, but non-significant differences (*p* > 0.05) were found (Figure 2b). However, there were significant differences (*p* = 0.0201) between the CDW and its corresponding medium (CDM), which indicated that the presence of wine-derived metabolites seemed to counteract the pro-leaky effects of the compounds present in the fermentation medium by itself. Additionally, the standard 3,4-dihydroxyphenylacetic acid (3,4-dhpa) at 200 µM (or 33.6 mg/L) reduced the paracellular transport of FITC-dextran in a statistically significant way (*p* < 0.001) (Figure 2b).

Numerous studies have reported protective effects on the paracellular permeability of phenolic-rich products using different cell models under inflammatory or homeostatic conditions [11,12,25,26,27]. This was the case for an aronia berry extract that significantly reduced cell permeability after 12 h of exposure (500–10,000 mg/L) to cytokine-stimulated Caco-2 cells [25]. A propolis extract tested in Caco-2 cells under non-inflammatory conditions decreased paracellular permeability in a dose-dependent manner (5–50 mg/L), although the effect was not significant [26]. In relation to wine polyphenols, Nunes et al. [12] observed a decrease in the paracellular permeability of a red wine extract (600 mg/L) in HT-29 colon epithelial cells stimulated or not by pro-inflammatory cytokines. However, in our study, using a dynamic gastrointestinal model to generate intestinal- and colonic-digested fluids and a Caco-2 cell model under homeostatic conditions to evaluate intestinal permeability, we have found non-significant effects on paracellular permeability by measuring the FITC-dextran paracellular transport. This disagreement with previous studies was explained in terms of quantitative differences in the phenolic solutions tested. In our study, the digested fluids (IDW and CDW) derived from a wine with a total phenolic content of 2500 mg/L. Bearing in mind that the fluids were previously diluted (1:40, *v*/*v*) to avoid cytotoxicity, the hypothetical total phenolic contents of the IDW- and CDW-diluted solutions in contact with cells were, respectively, 15.6 and 7.8 mg/L, which was in the concentration range used in other in vitro studies that found non-significant effects on paracellular permeability.

Another fact that should be taken into consideration in interpreting the results is the presence of ethanol in wine. Ethanol has been reported to increase permeability in people who consume alcohol excessively on a daily basis [28]. Wang et al. [29] specifically studied the effects of alcohol on intestinal epithelial barrier permeability and found that ethanol, even at the lowest concentration tested (1%) increased permeability to a certain extent. Bearing in mind the ethanol content of the wine selected for our study (13.5%) and the further dilutions, the ethanol contents of the IDW- and CDW-diluted solutions in contact with cells were calculated as 0.08% and 0.04%, respectively. Therefore, it was unlikely that the ethanol content in IDW and CDW would condition their effects on paracellular permeability.

Our results with quercetin (200 µM) concurred with previous studies that reported how quercetin enhanced cellular integrity in cell models, either in the absence or presence of stressful conditions of some kind. Among others, Suzuki and Hara [30] found a dose-dependent (10–100 μM) decrease in cell permeability using Caco-2 cells under homeostatic conditions. In another study, quercetin (10 µM) significantly improved cell permeability in Caco-2 cells treated or not treated with hydrogen peroxide to alter the cell monolayer [31]. With regard to 3,4-dhpa, to the best of our knowledge, this study is the first to report data about its potential effects on intestinal permeability, which reinforces the relevance of microbial-derived phenolic metabolites in intestinal homeostasis.

### 3.2. Effects of Digested-Wine Fluids on TJ Protein Expression

**Figure 3:**
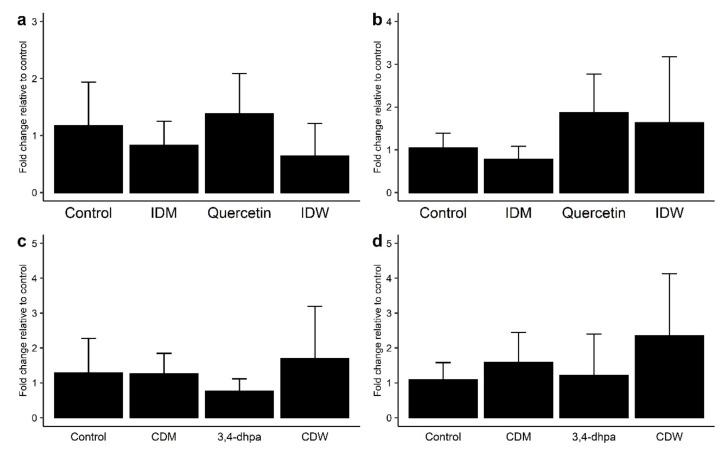
Effect on ZO-1 (**a,c**) and occludin (**b,d**) expression in Caco-2 cells of (**a**) intestinal digestion medium (IDM), quercetin (200 µM) and intestinal-digested wine (IDW) after 4 h of incubation, and (**b**) colon digestion medium (CDM), dihydroxyphenylacetic acid (200 µM) and colonic-digested wine (CDW) after 16 h of incubation.

As regards the genetic expression of TJ proteins, no changes in mRNA levels of ZO-1 with respect to the control were observed in the Caco-2 cell incubations with IDW (after 4 h of incubation) and CDW (after 16 h of incubation) (Figure 3a,c). However, slight increases in mRNA levels of occludin were found for both IDW and CDW fluids, although the differences were not statistically significant (*p* > 0.05) (Figure 3b,d). The same pattern was found for 200 µM quercetin (Figure 3a,b), whereas 3,4-dhpa exhibited almost no effects on the gene expression of either of both proteins (Figure 3c,d).

For the intestinal barrier to work properly, it requires the presence of TJ, which are central components of the barrier structure. Several studies have reported that polyphenols can also improve the synthesis and redistribution of TJ proteins, although results are quite variable [12,13,25,26,32,33,34]. Valdez et al. [25] reported the increase in ZO-1 protein expression after exposure of cytokine-stimulated Caco-2 cells to an aronia berry extract (500–10,000 mg/L), but no effect was observed on occluding expression. A propolis extract (5–50 mg/L) tested in Caco-2 cells under non-inflammatory conditions increased the expression of both ZO-1 and occludin, although the effect was not significant [26]. In the study of Nunes et al. [12], a significant increase in the expression of various TJ proteins (occludin, claudin and ZO-1) was observed after exposure of the wine extract (600 mg/L) to HT-29 cells, in the presence or absence of cytokines. Increases in occludin and ZO-1 expression also occurred after exposure of a grape seed extract (12.5 μg/mL) to LPS-stimulated Caco-2 cells [13]. In vivo, the administration to rats of a grape seed extract (water containing 0.1%, 21 days) produced an increase in the expression of occludin and ZO-1 in both the proximal and distal colon [34]. Our results may suggest a favorable effect of wine on the expression of occludin in Caco-2 cells grown under homeostatic conditions, but, as indicated above, the discrepancy with other studies was attributed to the relatively low phenolic concentration in the solutions tested (15.6 and 7.8 mg/L for IDW and CDW, respectively).

Therefore, under the in vitro conditions used in this study, wine seemed not to notably affect paracellular permeability or TJ protein expression, which did not discard a direct potential protective role in the intestinal barrier of wine polyphenols as seen for quercetin and its microbial-derived metabolite, 3,4-dihydroxyphenylacetic acid.

### 3.3. Phenolic Characterization of Digested-Wine Fluids

The phenolic characterization of the digested-wine fluids was carried out as an approach to determine the metabolic transformations that wine polyphenols suffer during digestion (Figure 1) and that might influence their effects on IP. Table 1 reports the individual phenolic content (mg/L) of the IDW and CDW fluids in comparison to the starting wine. Most of the compounds present in wine remained after simulated gastrointestinal digestion (IDW), with the exception of ellagic acid, tyrosol, esterified hydroxycinnamic acids (coutaric and caftaric acids), kaempferol, and some anthocyanins (malvidin-3-O-(6′′-acetyl) glucoside and petunidin-3-O-(6′′-*p*-coumaroyl) glucoside. On the other hand, 3-O-methygallic acid was found in IDW but was not present in wine (Table 1). Phenolic recovery percentages in IDW in comparison to the wine were largely dependent on chemical structure. Some phenolic acids such as protocatechuic (3,4-dihydroxybenzoic), 4-hydroxybenzoic and syringic (4-hydroxy-3,5-dimethoxy-benzoic) acids seemed not to be affected by gastrointestinal digestion as their IDW concentrations were around four times lower than those in the starting wine, which corresponded to the 1:4 (*v*/*v*) dilution inherent to the simulated gastrointestinal digestion. In contrast, gallic acid and its ethyl ester suffered a drastic reduction in their contents (around 1:600 and 1:17, respectively) after gastrointestinal simulation. Other compounds that showed reductions higher than 1:4 with respect to wine concentrations were: caffeic acid, ferulic acid, piceid, catechin, epicatechin, procyanidin B3, quercetin and its 3-O-glucoside, myricetin and its 3-O-rhamnoside, and some anthocyanins. Notably, compounds such as coumaric acid and resveratrol seemed to be released during gastrointestinal digestion as they showed a certain increase in their IDW concentrations in comparison to those in wine (1:1.9 for coumaric acid and 1:3 for resveratrol) (Table 1).

In relation to the colonic-digested wine (CDW), only 3-O-methylgallic acid, protocatechuic acid, and 4-hydroxybenzoic acid present in the previous intestinal-digested fluid (IDW) were found after simulated colonic fermentation (Table 1). Bearing in mind that the IDW fluid was 1:2 (*v*/*v*) diluted prior to its colonic fermentation, the concentration of protocatechuic acid slightly decreased after simulated colonic digestion (1:3.6 reduction with respect to the concentration values in IDW). In contrast, 3-O-methylgallic acid and 4-hydroxybenzoic acids seemed to be formed during colonic fermentation as they exhibited even higher CDW concentrations than those in the previous intestinal-digested fluid (1:0.89 and 1:0.85 for 3-O-methylgallic acid and 4-hydroxybenzoic acids, respectively) (Table 1). Notably, two compounds (4-hydroxyphenylacetic acid and 3,4-dihydroxyphenylacetic acid) were detected in the CDW but not in the previous intestinal-digested fluid. Their appearance was strictly related to the interaction of parent compounds with colonic microbiota.

Therefore, these results confirmed certain transformations of wine polyphenols during their in vitro digestion that might condition their bioactivity in the intestinal environment. For instance, 3-O-methylgallic acid, which appeared in the IDW fluid, was considered a product of the O-methylation of gallic acid and ethyl gallate after [35], which could explain, at least partly, their residual concentrations in the IDW fluid. The disappearance of coutaric acid after gastrointestinal digestion could be due to de-esterification reactions into the free acid (coumaric acid) [35], whose concentration was relatively higher in the IDW. The same could be said for the hydrolysis of piceid (resveratrol 3-O-β-d-glucoside) into free resveratrol that seemed to occur during gastrointestinal digestion. Indeed, it is in the colonic fermentation phase that wine polyphenols suffer most transformations leading to a large battery of phenolic metabolites such as those identified in the CDW fluid (3-O-methylgallic, protocatechuic, 4-hydroxybenzoic, 4-hydroxyphenylacetic, and 3,4-dihydroxyphenylacetic acids) [36]. The results are in line with previous studies concerning the colonic metabolism of wine polyphenols in vitro [15].

### 3.4. Effects of Digested-Wine Fluids on Fecal Microbiome and Microbial Metabolic Activity

As an indirect mechanism, wine polyphenols and/or their metabolites might specifically affect IP by means of modulating gut microbiota. Therefore, microbiome composition as well as microbial metabolic activity (i.e., production of short chain fatty acids [SCFAs]) during the wine colonic fermentations were determined (Figure 1).

As a first overview, Table 2 depicts the alpha diversity values (Observed, Shannon and Simpson indexes) for the fecal microbiota during their incubations in the absence (Fecal microbiota) and presence (Fecal microbiota + IDW) of the intestinal-digested wine up to 48 h. As expected from static fermentations, bacterial diversity tends to slightly decrease over time (24 and 48 h), with this response being more accentuated for the fermentations in the presence of IDW (Table 2). This could be due to the presence of some components of the IDW that could promote the growth of certain bacterial communities, contributing to a greater decrease in biodiversity compared to the evolution of the microbiota in the absence of IDW.

**Figure 4:**
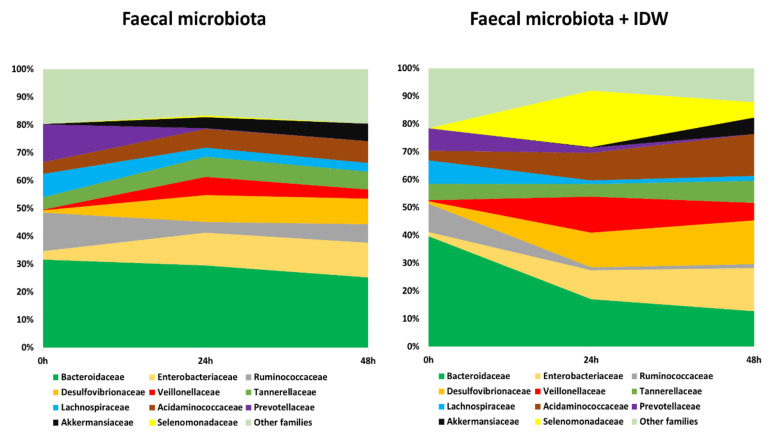
Evolution of bacterial families with relative abundance higher than 5% during fecal fermentations in the absence and in the presence of IDW (intestinal-digested wine).

In terms of bacterial taxonomy, *Bacteroidaceae*, *Ruminococcaceae*, *Prevotellaceae*, and *Lachnospiraceae* were the predominant families (higher percentage of relative abundance) in the initial stage of the fermentations (Figure 4, time 0 h). As fermentation progressed, the relative abundance of these bacterial families decreased, especially for *Bacteroidaceae* and *Ruminococcaceae* in the presence of IDW (Figure 4, times 24 and 48 h). In contrast, microbiota became enriched in *Enterobacteriaceae*, *Desulfovibrionaceae*, *Acidaminococcaceae*, *Veillonellaceae* and *Akkermansiaceae* families in both fermentations, in the presence and absence of IDW. A particular increase in the population of the *Selenomonadaceae* family was noted for the colonic fermentations at 24 and 48 h in the presence of IDW (Figure 4).

**Figure 5:**
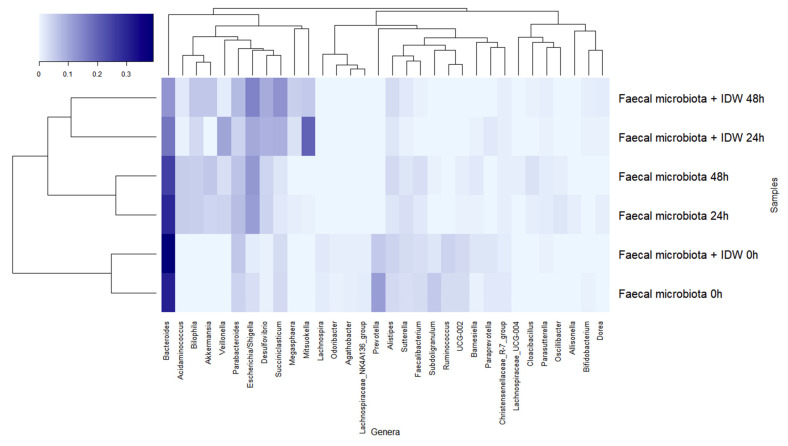
Evolution of bacterial genera with relative abundance higher than 1% during fecal fermentations in the absence and in the presence of intestinal-digested wine (IDW).

The dominant genera at the beginning of the fermentations were *Bacteroides*, *Prevotella*, and *Parabacteroides* (Figure 5). During fermentation, genera belonging to the families described above followed a similar pattern, leading to a reduction in *Bacteroides*, more rapidly in the presence of IDW, *Prevotella*, *Subdoligranulum*, and *Ruminococcus*, and an increase in *Veillonella*, *Escherichia*/*Shigella*, and *Akkermansia* in both fermentations, but the last two genera were more accentuated in the fermentations in the absence and presence of IDW, respectively. Additionally, microbiota in the fermentations in the presence of IDW increased in *Desulfovibrio*, *Megasphaera*, and *Mitsuokella*, while in the fermentations in its absence, the relative abundance of *Acidaminococcus* increased (Figure 5).

Overall, it should be noted that these results were obtained under static fermentation conditions which imply certain substrate depletion and might explain, for instance, the increase of *Proteobacteria* and *Akkermansia* observed for all fermentations and only obtained for one volunteer fecal microbiota. In spite of this, the presence of IDW was relevant enough to comparatively modify the fecal microbiome. The more pronounced reduction in *Bacteroides* proportions in the presence of IDW agrees with the results reported by Kemperman et al., in which reductions after the simulation with a red-wine extract in the SHIME gastrointestinal simulator were detected in members of this genus [37]. On the other hand, the increment in the proportions of the members of *Mitsuokella*, containing important protein degraders [38], as well as in *Desulfovibrio* and *Megasphaera* genera could be due to their ability to use different wine components or microbial-derived fermentation products as carbon source (e.g., lactate, acetate, amino acids) [39]; and their ability, in the case of some bacteria belonging to *Desulfovibrio* genus, to use them as electron donors for reduction of sulfate or other oxidized sulfur compounds to generate H_2_S [40]. In this sense, sulfate and sulfite are used as preservatives and antioxidants in wine, so all these conditions could provide an advantage to these microbial members, promoting their growth and hence the increase in their proportions in the overall microbiome.

The SCFA production during colonic fermentations in the absence and presence of IDW up to 48 h is shown in Table 3. As expected, SCFA concentrations increased during fecal fermentation, with acetic and propionic acids being the ones that exhibited the highest concentrations (Table 3). In general, this rise occurred mainly in the first 24 h, remaining almost stable until the end of the fermentation (48 h). When comparing fermentations in the absence and presence of IDW, significantly (*p* < 0.05) higher concentrations were found for propionic acid, the most abundant acid (Table 3). On the other hand, butyric acid, isovaleric acid and pentanoic acid showed significant lower concentrations when fecal microbiota were incubated in the presence of IDW (Table 3).

In any case, the changes in the SCFA production observed during IDW fecal fermentation were associated with the modulation of the microbiome by the digested-wine fluid. SCFA, mainly acetic, propionic, and butyric acids, are attracting considerable interest because of their possible importance for health and for the protection of the intestinal barrier integrity [41]. With regard to IP, a pioneering study by Mariadason et al. [42] first reported the capacity of SCFAs to reduce paracellular permeability in Caco-2 cells. Many other later studies have investigated the mechanisms surrounding this effect [43,44,45]. Regarding butyric and acetic acids, two well-known SCFAs derived from carbohydrate fermentation related to a protective effect on the intestinal barrier function by up-regulating tight junction protein claudin-1 or facilitating tight junction assembly [41,45], results revealed lower levels in the presence of IDW. It could be due to the decrease detected in the proportions of *Lachnospira*, *Ruminococcus*, and *Bacteroides* genus members, some of the main producers of these SCFA on gut microbiota [46]. In addition, it has been reported that some members of *Desulfovibrio*, *Megasphaera*, and *Mitsuokella* genera, that showed an increase after the fermentation in the presence of IDW, are able to ferment acetate and other acids such as lactate to produce mainly butyrate and H_2_S [40]. However, butyrate levels were lower in the presence of IDW, a fact that could be due the lower production of these SCFA by these microbial groups in comparison to *Ruminococcus* and *Bacteroides* members [47]. On the other hand, the branched-chain volatile fatty acid isovalerate, mainly produced during proteolytic fermentation of branched chain amino acids (valine, leucine, and isoleucine) by the intestinal microbiota [48], and the SCFA pentanoic acid, referred also as valeric acid, also revealed a significative decrease in the presence of IDW, a trend previously reported in subjects with a high adherence to the Mediterranean diet, rich, among others, in polyphenols [49,50,51]. This result could be due to the decrease detected in *Prevotella*, *Oscillospira, Bacteroides*, and *Alistipes* members, the main producers of isovaleric and valeric acids in the gut microbiota [47,52]. However, even though the levels of butyrate and acetate were lower in the presence of IDW, the expression of ZO-1 and Occludin on the Caco-2 cell line (Figure 3) seemed to increase in the presence of CDW. This might be associated with the higher levels of propionic acid production detected in the fermentation in presence of IDW, which, in turn, could be related to the increase in the levels of *Selenomonadaceae* family and *Akkermansia* and *Megasphaera* genus members in these conditions. An increment in propionic acid levels, whose production in our study was strongly stimulated in the presence of IDW, has been previously reported after the intake of grape/wine polyphenols and the adherence to the Mediterranean diet [50,51,53]. Furthermore, a recent study has reported that this acid promoted cell migration, inhibited activation of NLRP3 inflammasome, and maintained intestinal barrier function in LPS-induced IEC-6 cells [54]. Besides, some species belonging to the *Akkermansia* genus, such as *Akkermansia municiphila*, considered acetate and propionate producing bacteria [55], have been reported to enhance the expression of TJ proteins in Caco-2 cells [56].

Finally, the production of these SCFAs, together with other components present in the IDW fluid (mainly polyphenols) could also be contributing to the protective effect observed against the growth of some pathogen-related groups, such as *Veillonella* and *Escherichia*/*Shigella*, which showed lower levels in the presence of IDW in comparison to its absence. In this regard, the inhibitory effect of butyrate on the growth of opportunistic pathogens is well-known [57], as well as propionic acid, a fungicide and bactericide SCFA [58], and valerate, which, although they are less well-known SCFAs with limited research to date into their therapeutic potentials, have been related to an inhibitory effect against gut pathogens such as *Clostridioides difficile* and also appear to have an immunomodulatory effect [59,60]. Furthermore, it has also been proposed that butyrate and propionate play a role in alleviating the intestinal barrier dysfunction induced by pathogens’ LPS [43]. Therefore, taking this last into account, the increase in propionate production, probably due to the rise in *Akkermansia*, *Selenomonadaceae*, and *Megasphaera* levels, together with the phenolic metabolites present in the IDW, could be related to the lower increase in the proportions of pathogen-related groups in the presence of IDW as well as with the increase in ZO-1 and occludin tight junction proteins, decreasing paracellular permeability and improving and protecting the cell barrier.

## 4. Conclusions

This paper contributes with new evidence to the knowledge concerning the effects of polyphenols on intestinal health, and in particular on intestinal barrier integrity. Many mechanistic studies have reported the molecular effects on the intestinal permeability of dietary polyphenols without considering the metabolism reactions that they undergo within the human body. Therefore, one of the main inputs of this study has been to test digested-wine fluids obtained by using a well-standardized digestion model. Compositional analysis of the wine-derived intestinal- and colonic-digested fluids (IDW and CDW, respectively) confirmed active phenolic metabolism in both intestinal and colonic phases, in agreement with what is reported in the literature. Using a Caco-2 cell model under homeostatic conditions, non-significant effects on the paracellular permeability of the digested-wine fluids were observed, which was attributed to the relatively low phenolic concentration in the solutions tested. As regards the effects on TJ protein expression, slight increases in mRNA levels of occludin were observed for both IDW and CDW fluids, although without statistical significance. However, the fact that quercetin (present in wine and in IDW) and 3,4-dihydroxyphenylacetic acid (present in CDW) did lead to significant decreases in paracellular permeability in vitro led to us deciding not to discard a direct potential protective effect of whole polyphenols (and/or their metabolites) on the intestinal barrier in vivo. Another input of this paper has been to study the impact of wine-digested fluids on gut microbiota composition and functionality as an indirect mechanism to improve intestinal permeability. Results indicate that wine digestion promotes changes in the relative abundance of some families/genera as well as in the microbial production of SCFAs, all of which might affect paracellular permeability. Overall, this paper reinforces (wine) polyphenols as a promising dietary strategy to improve gut functionality and the relevance of microbial-derived phenolic metabolites in intestinal homeostasis.

## Figures and Tables

**Table 1:** Concentration of phenolic compounds (mg/L) in the wine and its intestinal-digested (IDW) and colon-digested (CDW) fluids.

	Wine	IDW	CDW
**Benzoic Acids and Derivatives**			
Gallic acid	115 ± 2	0.183 ± 0.003	nd
Ethyl gallate	9.20 ± 0.46	0.537 ± 0.048	nd
3-O-methygallic acid	nd	0.775 ± 0.042	0.875 ± 0.170
Protocatechuic acid	5.40 ± 0.03	1.49 ± 0.07	0.410 ± 0.029
4-Hydroxybenzoic acid	0.843 ± 0.029	0.218 ± 0.003	0.256 ± 0.039
Vanillic acid	3.56 ± 0.21	traces	nd
Syringic acid	5.24 ± 0.82	1.35 ± 0.03	nd
Ellagic acid	12.4 ± 2.4	nd	nd
**Benzoic Alcohols**			
Tyrosol	121 ± 5	nd	nd
**Hydroxycinnamic Acids and Derivatives**			
Caffeic acid	12.8 ± 0.6	1.27 ± 0.049	nd
Coumaric acid	4.92 ± 2.05	2.59 ± 0.078	nd
Ferulic acid	0.281 ± 0.050	traces	nd
Coutaric acid	10.1 ± 0.4	nd	nd
Caftaric acid	4.27 ± 0.51	nd	nd
**Stilbenes**			
Resveratrol	1.69 ± 0.06	0.554 ± 0.012	nd
Piceid	8.53 ± 1.94	0.493 ± 0.013	nd
**Flavan-3-ols**			
(+)-Catechin	16.2 ± 2.7	0.105 ± 0.048	nd
(−)-Epicatechin	9.68 ± 1.31	0.115 ± 0.048	nd
Procyanidin B1	1.01 ± 0.10	0.442 ± 0.029	nd
Procyanidin B3	0.018 ± 0.024	traces	nd
**Flavonols**			
Quercetin	3.97 ± 0.19	traces	nd
Myricetin	4.47 ± 0.37	traces	nd
Kaempferol	0.231 ± 0.061	nd	nd
Quercetin-3-O-glucoside	4.42 ± 0.09	0.031 ± 0.004	nd
Myricetin 3-O-rhamnoside	0.272 ± 0.052	traces	nd
**Anthocyanins**			
Delphinidin-3-*O*-glucoside	0.121 ± 0.001	traces	nd
Cyanidin-3-*O*-glucoside	traces	traces	nd
Petunidin-3-*O*-glucoside	0.214 ± 0.002	traces	nd
Peonidin-3-*O*-glucoside	0.262 ± 0.004	traces	nd
Malvidin-3-*O*-glucoside	2.85 ± 0.01	0.0299 ± 0.0011	nd
Malvidin-3-*O*-(6′′-acetyl)glucoside	traces	nd	nd
Petunidin-3-*O*-(6′′-*p*-coumaroyl)glucoside	traces	nd	nd
Peonidin-3-*O*-(6′′-*p*-coumaroyl)glucoside	traces	traces	nd
Malvidin-3-*O*-(6′′-*p*-coumaroyl)glucoside	0.145 ± 0.003	traces	nd
**Hydroxyphenylacetic Acids**			
4-hydroxyphenylacetic acid	nd	nd	0.432 ± 0.042
3,4-dihydroxyphenylacetic acid	nd	nd	0.630 ± 0.031

nd: not detected.

**Table 2:** Different alpha diversity indices (Observed, Shannon, and Simpson) during the fecal fermentations in the absence or presence of the intestinal-digested wine (IDW).

		0 h	24 h	48 h
Observed	Fecal microbiota	221	227	194
	Fecal microbiota + IDW	213	148	143
Shannon	Fecal microbiota	4.25	3.98	3.95
	Fecal microbiota + IDW	4.15	3.78	3.66
Simpson	Fecal microbiota	0.966	0.959	0.959
	Fecal microbiota + IDW	0.963	0.961	0.946

**Table 3:** Production of SCFAs (µM) during fecal fermentations in the absence and in the presence of intestinal-digested wine (IDW). Data are expressed as means ± SD of the concentration of SCFAs in the three fermentations.

	Fecal Microbiota			Fecal Microbiota + IDW		
	0 h	24 h	48 h	0 h	24 h	48 h
Acetic acid	1.67 ± 0.43	13.8 ± 1.6	14.3± 2.3	2.16 ± 0.42	11.5 ± 0.7	13.3 ± 1.2
Propionic acid	nd	3.37 ± 0.26	2.56 ± 0.55	nd	15.3 ± 1.4 *	12.9 ± 2.0 *
Butyric acid	0.34 ± 0.59	9.53 ± 0.65	9.42 ± 0.95	0.23 ± 0.48	4.77 ± 0.84 *	6.36 ± 0.41 *
Isovaleric acid	nd	2.94 ± 0.13	3.04 ± 0.31	nd	1.27 ± 0.21 *	1.57 ± 0.16 *
Pentanoic acid	nd	8.13 ± 1.07	12.7 ± 0.8	nd	6.17 ± 0.72 *	8.52 ± 0.32 *

nd = not detected. * significant differences at *p* < 0.05 with respect to the absence of IDW at the same incubation time.

## Data Availability

The data presented in this study are available on request from the corresponding author.
